# Magnetic resonance three-dimensional steady-state free precession imaging of the thoracic duct in patients with Fontan circulation and its relationship to outcomes

**DOI:** 10.1186/s12968-023-00937-w

**Published:** 2023-06-12

**Authors:** Daniel A. Castellanos, Sidra Ahmad, Nicole St. Clair, Lynn A. Sleeper, Minmin Lu, David N. Schidlow, Rahul H. Rathod, Suellen M. Yin, Jesse J. Esch, David Annese, Andrew J. Powell, Luis Quiñonez, Raja Shaikh, Sunil J. Ghelani

**Affiliations:** 1 Department of Cardiology, Boston Children’s Hospital, 300 Longwood Ave, BCH 3215, Boston, MA 02115 USA; 2 Department of Pediatrics, Harvard Medical School, Boston, MA USA; 3 Department of Cardiac Surgery, Boston Children’s Hospital and Harvard Medical School, Boston, MA USA; 4 Department of Radiology, Boston Children’s Hospital and Harvard Medical School, Boston, MA USA

**Keywords:** Congenital heart disease, Fontan operation, Thoracic duct, Lymphangiography, Magnetic resonance imaging

## Abstract

**Background:**

Lymphatic complications are common in patients with Fontan circulation. Three-dimensional balanced steady-state free precession (3D bSSFP) angiography by cardiovascular magnetic resonance (CMR) is widely used for cardiovascular anatomical assessment. We sought to determine the frequency of thoracic duct (TD) visualization using 3D bSSFP images and assess whether TD characteristics are associated with clinical outcomes.

**Methods:**

This was a retrospective, single-center study of patients with Fontan circulation who underwent CMR. Frequency matching of age at CMR was used to construct a comparison group of patients with repaired tetralogy of Fallot (rTOF). TD characteristics included maximum diameter and a qualitative assessment of tortuosity. Clinical outcomes included protein-losing enteropathy (PLE), plastic bronchitis, listing for heart transplantation, and death. A composite outcome was defined as presence of any of these events.

**Results:**

The study included 189 Fontan patients (median age 16.1 years, IQR 11.0–23.2 years) and 36 rTOF patients (median age 15.7 years, IQR 11.1–23.7 years). The TD diameter was larger (median 2.50 vs. 1.95 mm, p = 0.002) and more often well visualized (65% vs. 22%, p < 0.001) in Fontan patients vs. rTOF patients. TD dimension increased mildly with age in Fontan patients, R = 0.19, p = 0.01. In Fontan patients, the TD diameter was larger in those with PLE vs. without PLE (age-adjusted mean 4.11 vs. 2.72, p = 0.005), and was more tortuous in those with NYHA class ≥ II vs. class I (moderate or greater tortuosity 75% vs. 28.5%, p = 0.02). Larger TD diameter was associated with a lower ventricular ejection fraction that was independent of age (partial correlation = − 0.22, p = 0.02). More tortuous TDs had a higher end-systolic volume (mean 70.0 mL/m^2^ vs. 57.3 mL/m^2^, p = 0.03), lower creatinine (mean 0.61 mg/dL vs. 0.70 mg/dL, p = 0.04), and a higher absolute lymphocyte count (mean 1.80 K cells/µL vs. 0.76 K cells/µL, p = 0.003). The composite outcome was present in 6% of Fontan patients and was not associated with TD diameter (p = 0.50) or tortuosity (p = 0.09).

**Conclusions:**

The TD is well visualized in two-thirds of patients with Fontan circulation on 3D-bSSFP images. Larger TD diameter is associated with PLE and increased TD tortuosity is associated with an NYHA class ≥ II.

**Supplementary Information:**

The online version contains supplementary material available at 10.1186/s12968-023-00937-w.

## Introduction

Systemic venous hypertension in the Fontan circulation results in lymphatic abnormalities, which are increasingly recognized [1]. These abnormalities underlie the pathophysiology of Fontan complications such as protein losing enteropathy (PLE) and plastic bronchitis [2]. While invasive magnetic resonance lymphangiography after injection of gadolinium-based contrast agents in the lymphatic system is ideal for definitive visualization and intervention planning [3, 4], non-invasive techniques often clearly depict larger lymphatic channels such as the thoracic duct [5, 6]. In patients with single ventricle physiology, a greater severity of lymphatic abnormality as seen on non-invasive T2-weighted (T2W) sequences was associated with patient-related outcomes [7–9]. Three-dimensional (3D) whole-heart cardiovascular magnetic resonance (CMR) using balanced steady-state free precession (bSSFP) is a widely adopted tool for the anatomical assessment of congenital heart disease. While the primary goal of this sequence is to image arterial and venous anatomy, the thoracic duct can be visualized [10]. This may allow for anatomic characterization of the thoracic duct, such as size and tortuosity. These thoracic duct characteristics have only been reported in small numbers of patients with Fontan circulation and a comprehensive assessment of thoracic duct size and tortuosity using 3D bSSFP in patients with Fontan circulation has not been reported [1, 11].

The aim of this study is to determine the frequency of visualization of the thoracic duct using a 3D bSSFP sequence in patients with Fontan circulation and in patients with repaired tetralogy of Fallot (rTOF) with CMR examinations obtained at similar average age. Additionally, this study assesses how thoracic duct characteristics (size and tortuosity) are associated with clinical outcomes in patients with Fontan circulation. The hypothesis is that the thoracic duct will more often be completely or nearly completely visualized in patients with Fontan circulation compared with patients with rTOF, and larger or more tortuous thoracic ducts will be associated with worse clinical outcomes.

## Methods

### Study population and inclusion criteria

This was a retrospective, single-center, study of patients with Fontan circulation who underwent CMR with 3D bSSFP imaging at Boston Children’s Hospital between January 1, 2011 and November 1, 2021. Typical imaging parameters for the 3D bSSFP whole-heart angiogram in an adult patient are as follows: field of view 410 × 240 mm, voxel size 1.6 × 1.6 × 1.6 mm^3^ reconstructed to 0.6 × 0.6 × 0.6 mm^3^, flip angle 110°, echo time 2.2 ms, repetition time 4.5 ms, and compressed SENSE reduction factor 3. Respiratory motion compensation was performed using a self-navigator to track and gate heart position [12]. In the cases of multiple CMR examinations per patient, only the most recent study was included. Frequency matching with respect to age at CMR was used to construct a comparison group of patients with repaired TOF. Other clinical data reviewed included clinical notes, exercise stress tests, cardiac catheterizations, and echocardiograms obtained within 1 year of CMR.

**Fig. 1:**
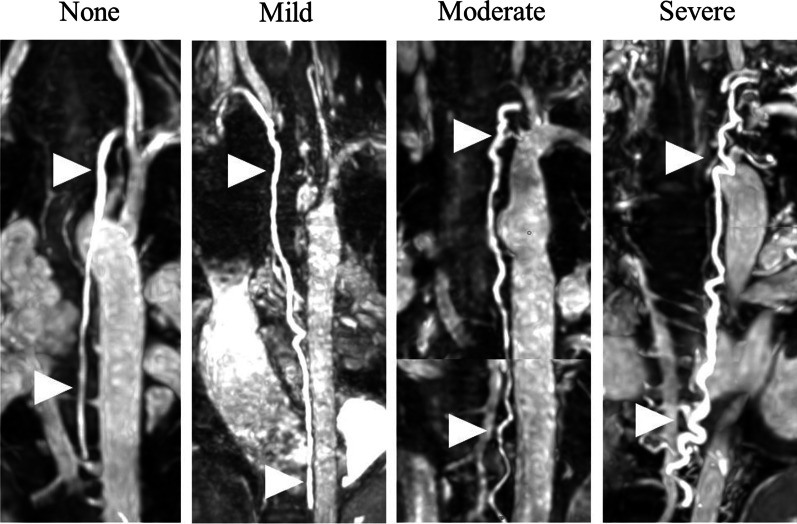
Representative examples of the subjective thoracic duct tortuosity grading scale used for this project. Arrowheads indicate the thoracic duct. The following guidance was used when assigning tortuosity: no turns/deviations from course for no tortuosity, one turn or multiple very subtle turns for mild tortuosity, more than one large turn or multiple small turns for moderate tortuosity, and turns along the entire or nearly entire course for severe tortuosity. The images for moderate and severe grading are a composite of two reformatted images to best show the course of the thoracic duct

Thoracic duct visualization was classified as completely visualized, nearly completely visualized, incompletely visualized, or not visualized. A completely visualized thoracic duct is one in which the entire length through the thorax to its insertion at the venous angle can be identified. A nearly completely visualized thoracic duct is one in which only a short length (no greater than the length of two vertebrae) is not visualized. Larger portions of the thoracic duct are not well visualized in an incompletely visualized thoracic duct. Thoracic duct size was represented by the maximum thoracic duct diameter. To obtain the maximum thoracic duct diameter the largest portion of the thoracic duct was first identified in the coronal plane. Then, the largest diameter of that area was obtained in a short axis (double oblique) view. Thoracic duct tortuosity was qualitatively graded as none, mild, moderate, and severe based on predefined representative cases (Fig. 1) and the following guidance: no turns/deviations from course for no tortuosity, one turn or multiple very subtle turns for mild tortuosity, more than one large turn or multiple small turns for moderate tortuosity, and turns along the entire or nearly entire course for severe tortuosity. In patients in which a T2-weighted (T2W) sequence for lymphatic assessment was obtained, the T2W sequence was reviewed to assess whether the identified the thoracic duct on the 3D bSSFP sequence corresponded with the thoracic duct visualized on the T2W sequence. Patients were classified by New York Heart Association (NYHA) functional class based on review of the clinical notes. Aortopulmonary collateral burden was represented as a percent of systemic blood flow and was calculated using the systemic method [13]. Exercise test results were only included if peak respiratory exchange ratio was greater than 1.09 (a threshold for peak exertion in our lab). A composite clinical outcome was defined as the presence of PLE, plastic bronchitis, listing for heart transplantation, or death. The study was approved by the Institutional Review Board at Boston Children’s Hospital and the requirement for informed consent was waived.

### Statistical analysis

Thoracic duct visualization was dichotomized as completely or nearly completely visualized versus incompletely or not visualized. For all analyses except for interobserver and intraobserver agreement, thoracic duct tortuosity was analyzed as a dichotomous variable: none or mild versus moderate or severe. Descriptive statistics are presented as mean ± standard deviation (SD) for continuous measures with approximately a normal distribution and median (interquartile range) for other continuous or ordinal measures. Categorical data are described as frequency and percentage. Where mean comparisons are presented, Student’s t-test or ANOVA was performed and where median comparisons are presented, a Wilcoxon rank sum test or Kruskal–Wallis test was performed. For comparisons of categorical variables, a Fisher exact or Chi-square test was performed.

Analysis of covariance was also used to estimate mean thoracic duct size adjusted for differences in age at CMR in patients with Fontan circulation with vs. without clinical outcome. Multiple linear regression was used to estimate the association between thoracic duct size and clinical variables while controlling for age. A second observer repeated measurements for thoracic duct visualization, tortuosity, and maximum diameter in 50 patients. The first observer repeated measurements for thoracic duct tortuosity and maximum diameter in 50 patients 1 year after initial data collection to assess intraobserver agreement. For thoracic duct visualization and tortuosity (analyzed as a categorical variable with 4 categories), agreement was assessed using percentage agreement and a kappa statistic. For thoracic duct diameter, agreement was assessed using the intraclass correlation coefficient.

A p-value < 0.05 was considered to be statistically significant. Analyses were performed with SAS 9.4 (SAS Institute, Inc., Cary, North Carolina) and R version 4.03 (R Core Team (2020). R: A language and environment for statistical computing. R Foundation for Statistical Computing, Vienna, Austria. URL https://www.R-project.org/).

## Results

**Fig. 2:**
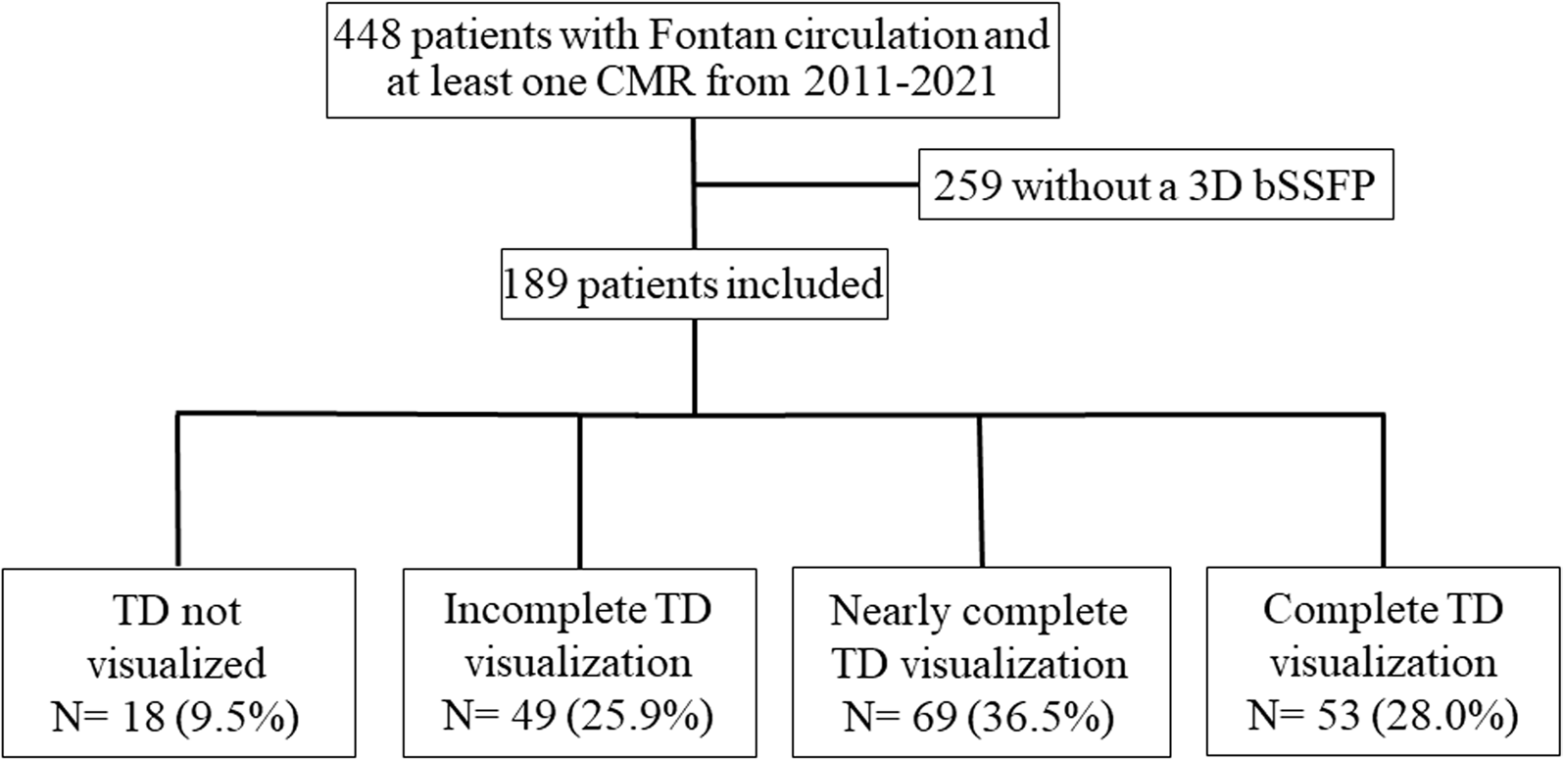
The study population was defined as all patients with Fontan circulation and a cardiac MRI that included 3D bSSFP imaging. Of the 448 patients with Fontan circulation who had a cardiac MRI during the study period, 189 had a 3D bSSFP and the thoracic duct was completely or nearly completely visualized in 122 of these patients. 3D: Three-dimensional, bSSFP: balanced steady state free precession, TD: thoracic duct

**Fig. 3:**
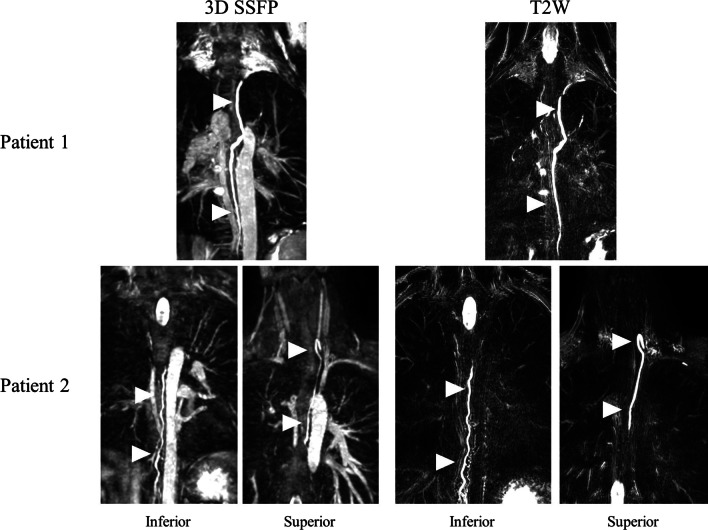
Comparison of thoracic duct visualization between 3D bSSFP and T2W lymphatic imaging in two patients. In each of the 29 patients with Fontan circulation who also had T2W lymphatic imaging the thoracic duct as visualized on 3D bSSFP matched the thoracic duct as visualized on T2W. Arrowheads indicate the thoracic duct

Of the 448 patients with Fontan circulation who had a CMR examination during the study period, 189 patients met the inclusion criteria (Fig. 2). The group of patients with Fontan circulation was 64% male and 20% of the patients had heterotaxy syndrome (Additional file 1: Table S1). The cardiac diagnoses included 33% with hypoplastic left heart syndrome, 18% with tricuspid atresia, 17% with double outlet right ventricle, 10% with double inlet left ventricle, and 7% with an unbalanced complete atrioventricular canal. A lateral tunnel Fontan operation was performed in 66% and an extracardiac conduit Fontan operation was performed in 24%. CMR examinations from 36 patients with rTOF were reviewed. The median age at CMR was 16.1 years (IQR 11.0–23.2 years) in patients with Fontan circulation and 15.7 years (IQR 11.1–23.7 years) in patients with rTOF. In each of the 29 patients with Fontan circulation who also had T2W lymphatic imaging, the thoracic duct as visualized on 3D bSSFP matched the thoracic duct as visualized on T2W (Fig. 3).

### Comparison between patients with Fontan circulation and patients with rTOF

Between the groups with Fontan circulation and rTOF, there was no difference in sex, age, or body surface area (BSA) at CMR (Table 1). The thoracic duct was better seen (complete or nearly complete visualization in 65% vs. 22%, p < 0.001) in patients with Fontan circulation relative to patients with rTOF. There was no difference between the frequencies of image artifact between the two groups (9% vs. 11%, p = 0.75, Table 1). In patients with Fontan circulation, the thoracic duct was more often well seen in male patients (Additional file 1: Table S2). There was no statistically significant difference in visualization based on the primary cardiac diagnosis, type of Fontan, ventricular morphology, age, or BSA.

**Table 1:** Comparison of patients with Fontan circulation and rTOF

	Fontan circulation (n = 189)	rTOF (n = 36)	p-value
Male	121 (64%)	17 (47%)	0.06
Median age at CMR, yrs	16.1 (11.0, 23.2)	15.7 (11.1, 23.7)	0.86
Mean BSA at CMR, m^2^	1.49 ± 0.44	1.49 ± 0.39	1.00
Thoracic duct visualization			**< 0.001**
Complete/Nearly complete	122 (65%)	8 (22%)	
Incomplete/Not visualized	67 (35%)	28 (79%)	
Maximum thoracic duct diameter, mm	2.50 (2.10, 3.10)	1.95 (1.70, 2.60)	**0.002**
Thoracic duct tortuosity			0.17
None/Mild	119 (63%)	17 (47%)	
Moderate/Severe	50 (27%)	3 (8%)	
3D sequence with artifact or of poor quality	17 (9%)	4 (11%)	0.75
3D sequence with limited field of view	6 (3%)	0 (0%)	0.59

Values are count (%), median (IQR), or mean ± standard deviation. Bold indicates a p-value < 0.05. *rTOF* repaired tetralogy of Fallot, *CMR* cardiovascular magnetic resonance, *BSA* body surface area, *FOV* field of view

Contrast was administered to 84 (44%) patients with a Fontan circulation. Gadobutrol (Gadavist^®^) was administered to 76 patients, gadobenate dimeglumine (Magnevist^®^) to 7 patients, and gadofosveset trisodium (Ablavar^®^) to one patient. At our institution, if a patient receives contrast it is routinely administered prior to acquisition of the 3D bSSFP sequence. There was no difference in thoracic duct visualization rates between those who received contrast and those who did not receive contrast (complete or nearly complete visualization of 69% in those that did receive contrast vs. 61% in those that did not, p = 0.29).

The thoracic duct diameter was measured in 171 patients with Fontan circulation and 20 patients with rTOF. In patients with rTOF, thoracic duct diameter did not correlate with BSA, with a Spearman’s rank-order correlation of *r*_s_(18) = − 0.034, p = 0.89. Therefore, unadjusted mean or median diameters are reported when comparing these two groups of patients. The thoracic duct was larger (median diameter of 2.50 mm IQR 2.10, 3.10 mm vs. 1.95 mm IQR 1.70, 2.60 mm, p = 0.002) in patients with Fontan circulation relative to patients with rTOF (Table 1). Thoracic duct tortuosity was assigned to 169 patients with Fontan circulation and 20 patients with rTOF. There was no difference in thoracic duct tortuosity between patients with Fontan circulation and patients with rTOF (moderate or severe tortuosity in 27% vs. 8%, p = 0.17). While the primary cardiac diagnosis and type of Fontan pathway were different, thoracic duct tortuosity did not vary by sex, age at Fontan operation, need for Fontan revision, age at MRI, BSA, or the presence of heterotaxy syndrome (Table 2).

**Table 2:** Characteristics of patients with Fontan circulation by thoracic duct tortuosity

Variable	None/mild (n = 119)	Moderate/severe (n = 50)	p-value
Male	78 (66%)	33 (66%)	1.00
Primary cardiac diagnosis			**0.05**
Tricuspid atresia	24 (20%)	6 (12%)	
HLHS	39 (33%)	18 (36%)	
DILV	10 (8%)	6 (12%)	
DORV	25 (21%)	3 (6%)	
Unbalanced CAVC	6 (5%)	4 (8%)	
Small right heart	6 (5%)	3 (6%)	
Other	9 (8%)	10 (20%)	
Heterotaxy syndrome			1.00
Yes	22 (19%)	9 (18%)	
No	97 (82%)	41 (82%)	
Median age at Fontan, years	2.7 (2.2, 3.6)	3.0 (2.3, 3.7)	0.23
Fontan type			**0.04**
Lateral tunnel	86 (72%)	26 (52%)	
Extracardiac	25 (21%)	17 (34%)	
RA-PA	7 (6%)	5 (10%)	
Other	1 (1%)	2 (4%)	
Fontan revision			1.00
Yes	5 (4%)	2 (4%)	
No	114 (96%)	48 (96%)	
Ventricular morphology			0.08
Right	57 (50%)	20 (42%)	
Left	44 (38%)	15 (31%)	
Both	14 (12%)	13 (27%)	
Median age at MRI, years	17.1 (12.3, 24.5)	15.2 (10.4, 22.6)	0.27
BSA at MRI	1.54 ± 0.42	1.46 ± 0.49	0.32

Values are count (%), median (IQR), or mean ± standard deviation. Bold indicates a p-value < 0.05. *HLHS* hypoplastic left heart syndrome, *DILV* double inlet left ventricle, *DORV* double outlet right ventricle, *CAVC* complete atrioventricular canal, *RA* right atrium, *PA* pulmonary artery, *BSA* body surface area

### Clinical variables and thoracic duct characteristics in patients with Fontan circulation

Clinical events in patients with Fontan circulation are demonstrated in Table 3. In the patients with Fontan circulation, 1% had plastic bronchitis (in each case it was diagnosed prior to CMR), 2% had PLE (half were diagnosed prior to CMR), 11% had NYHA class ≥ II, 9% had a major thrombotic event, and 28% had a history of arrhythmia (23% with an atrial arrhythmia, 2% with both an atrial and ventricular arrhythmia). Thoracic duct diameter increased mildly with age in patients with Fontan circulation, R = 0.19, p = 0.01, and age at CMR differs according to outcome status for several of the outcomes. Therefore, age-adjusted means with standard error (SE) were reported. The thoracic duct diameter was larger in patients with PLE (age-adjusted mean of 4.11 SE 0.49 mm vs. 2.72 SE 0.07 mm, p = 0.005) vs. patients without PLE, and was more tortuous in patients with NYHA class ≥ II vs. patients with NYHA class I (moderate or severe tortuosity in 55% vs. 26%, p = 0.02).

**Table 3:** Age-adjusted thoracic duct diameter, and tortuosity by clinical event in patients with Fontan circulation (N = 189)

Clinical event	Event	No event	p-value
Plastic bronchitis, n	2	187	
Age at CMR, years	7.1 ± 1.4	18.5 ± 10.0	0.11
Maximum thoracic duct diameter, mm	2.8 (0.7)	2.8 (0.1)	0.95
Tortuosity			0.51
None/mild	1 (50%)	118 (71%)	
Moderate/severe	1 (50%)	49 (29%)	
PLE, n	4	185	
Age at CMR, years	12.9 ± 3.9	18.5 ± 10.1	0.27
Maximum thoracic duct diameter, mm	4.1 (0.5)	2.7 (0.1)	**0.005**
Tortuosity			0.08
None/mild	1 (25%)	118 (72%)	
Moderate/severe	3 (75%)	47 (29%)	
NYHA class ≥ II, n	21	168	
Age at CMR, years	21.1 ± 11.7	18.0 ± 9.8	0.19
Maximum thoracic duct diameter, mm	2.9 (0.2)	2.7 (0.1)	0.50
Tortuosity			**0.02**
None/mild	9 (45%)	110 (74%)	
Moderate/severe	11 (55%)	39 (26%)	
Major thrombotic event, n	17	166	
Age at CMR, years	23.4 ± 17.0	17.9 ± 9.0	0.20
Maximum thoracic duct diameter, mm	2.4 (0.3)	2.8 (0.01)	0.13
Tortuosity			1.00
None/mild	11 (73%)	102 (69%)	
Moderate/severe	4 (27%)	46 (31%)	
Arrhythmia history, n	52	137	
Age at CMR, years	23.6 ± 12.8	16.3 ± 8.0	**< 0.001**
Maximum thoracic duct diameter, mm	2.5 (0.2)	2.8 (0.1)	0.12
Tortuosity			0.35
None/mild	35 (76%)	84 (68%)	
Moderate/severe	11 (24%)	39 (32%)	

Values are mean ± standard deviation, age-adjusted mean (standard error), or count (%). Given thoracic duct dimension increases mildly with age, the age-adjusted mean is presented. Bold indicates a p-value < 0.05. CMR: Cardiovascular magnetic resonance. FOV: Field of view. PLE: Protein losing enteropathy, NYHA: New York Heart Association

Of the 189 patients with Fontan circulation, 51 (27%) had a fenestration, 130 (69%) did not have a fenestration, and 8 (4%) had an unknown fenestration status. Age at CMR was significantly younger in the fenestration group (mean age of 14.4 ± 8.6 vs. 20.0 ± 10.0 years, p < 0.001). After adjusting for age, mean thoracic dimension was not significantly different based on fenestration status (age-adjusted mean of 2.58 SE 0.15 mm vs. 2.82 SE 0.09 mm in those with vs. without a fenestration, p = 0.18). There was no difference in the frequency of moderate or severe thoracic duct tortuosity between those with and without a fenestration (moderate or severe tortuosity in 31% with a fenestration vs. 27% without a fenestration, p = 0.70).

Patients with more tortuous thoracic ducts had a higher indexed ventricular end-systolic volume (mean of 70.0 ± 29.9 mL/m^2^ vs. 57.3 ± 2.60 mL/m^2^, p = 0.03), lower creatinine (mean of 0.61 ± 0.22 mg/dL vs. 0.70 ± 0.20 mg/dL, p = 0.04), and a higher absolute lymphocyte count (mean of 1.80 ± 0.78 K cells/µL vs. 0.76 ± 0.36 K cells/µL, p = 0.003, Table 4). Thoracic duct size was inversely correlated with ejection fraction independent of age (controlling for age), although the magnitude was small (partial correlation = − 0.22, p = 0.02, Table 5). Four patients were listed for heart transplantation (three were listed prior to CMR, one patient eventually underwent heart transplant) and four patients died. The median time from CMR to last date of clinical follow-up was 10.8 months (IQR 0.1, 27.0 months). Thoracic duct size (age-adjusted mean of 2.04 SE 0.50 mm vs. 2.77 SE 0.08 mm in those who died vs. survived, p = 0.149) and tortuosity (moderate or severe tortuosity in 25% vs. 30% in those who died vs. survived, p = 1.00) were not associated with death. The composite outcome was present in 11 (6%) of patients with Fontan circulation (mean age of 18.8 ± 12.8 years in those with the composite outcome and 18.3 ± 9.9 years in those without the composite outcome, p = 0.87) and was not associated with thoracic duct diameter (age-adjusted mean of 2.94, standard error (SE) 0.30 mm vs. 2.73 SE 0.08 mm in those with severe complication vs. without severe complication, p = 0.50) or tortuosity (moderate or severe tortuosity in 55% vs. 28% in those with severe complication vs. without severe complication, p = 0.09). Thoracic duct visualization and tortuosity showed substantial inter- and intraobserver agreement and maximum thoracic duct diameter showed moderate to good agreement (Table 6) [14, 15].

**Table 4:** Clinical measures by thoracic duct tortuosity in patients with Fontan circulation (N = 169)

Variable	None/mild (n = 119)		Moderate /severe (n = 50)		p-value
	n	Value	n	Value	
CMR					
EDV/BSA, ml/m^2^	86	116 ± 33.9	35	132.3 ± 45.7	0.06
ESV/BSA, ml/m^2^	84	57.3 ± 26.0	32	70.0 ± 29.9	**0.03**
Mass/BSA, g/m^2^	77	64.5 ± 22.7	28	75.6 ± 37.0	0.15
EF, %	84	52.4 ± 8.6	32	49.2 ± 8.4	0.09
APC, %	20	16.8 ± 9.6	8	21.0 ± 9.5	0.31
Moderate or worse AVVR (echo)	85	9 (11%)	39	7 (18%)	0.26
Cath					
Mean Fontan pressure, mmHg	27	15.0 ± 3.0	18	16.5 ± 3.6	0.14
PVR, WUm^2^	26	2.16 ± 0.77	17	1.72 ± 0.83	0.09
Max EDP, mmHg	26	8.96 ± 2.60	17	10.53 ± 2.65	0.06
CPET					
Peak RER	34	1.20 ± 0.09	11	1.18 ± 0.09	0.42
Peak VO_2_, mL × kg^−1^ × min^−1^	34	24.1 ± 7.2	11	20.8 ± 7.2	0.19
% predicted VO_2_ max	34	62.4 ± 14.0	11	58.6 ± 16.0	0.46
% predicted peak O_2_ pulse	34	76.5 ± 20.9	11	72.0 ± 22.1	0.54
% predicted peak work rate	33	71.5 ± 17.8	11	65.2 ± 23.3	0.35
Relative VO_2_ at VAT mL × kg^−1^ × min^−1^	34	14.3 ± 3.9	11	13.7 ± 4.5	0.69
%VAT/Predicted peak VO_2_	34	37.3 ± 7.9	11	39.3 ± 12.6	0.63
VE/VCO_2_ slope	34	35.4 ± 7.5	11	30.9 ± 7.4	0.09
LAB					
Creatinine, mg/dL	52	0.70 ± 0.20	35	0.61 ± 0.22	**0.04**
Albumin, mg/dL	49	4.4 ± 0.6	33	4.4 ± 0.5	0.71
Absolute Lymphocyte Count, K cells/µL	13	0.76 ± 0.36	9	1.80 ± 0.78	**0.003**

Values are mean ± standard deviation or count (%). Bold indicates a p-value < 0.05. CMR: cardiovascular magnetic resonance. Cath: cardiac catheterization. *CPET* cardiopulmonary exercise testing, *LAB* laboratory values, *FOV* field of view, *EDV* end-diastolic volume, *BSA* body surface area, *ESV* end-systolic volume, *EF* ejection fraction, *APC* aortopulmonary collateral burden, *AVVR* atrioventricular valve regurgitation, *echo* echocardiography, *PVR* pulmonary vascular resistance, *EDP* end-diastolic pressure, *RER* respiratory exchange ratio, *VO*_*2*_ oxygen consumption, *VAT* ventilatory anaerobic threshold, *VE* ventilation

**Table 5:** Partial correlation of clinical measures to maximum thoracic duct diameter in patients with Fontan circulation, controlling for age (N = 171)

Variable	Mean ± SD	Partial correlation		
		n	R	p-value
CMR				
Years since initial Fontan	14.6 ± 8.8	171	− 0.08	0.30
EDV/BSA, ml/m^2^	120.9 ± 37.4	122	0.06	0.51
ESV/BSA, ml/m^2^	60.9 ± 27.1	117	0.13	0.16
Mass/BSA, g/m^2^	67.4 ± 27.3	106	0.02	0.81
EF, %*	51.4 ± 8.4	117	− 0.22	**0.02**
APC, %	18.5 ± 9.8	29	− 0.19	0.32
Cath				
Mean Fontan pressure, mmHg	15.6 ± 3.3	46	0.16	0.30
PVR, WUm^2^	2.0 ± 0.8	44	− 0.16	0.29
Max EDP, mmHg	9.6 ± 3.0	44	0.28	0.07
CPET				
Peak VO_2_, mL × kg^−1^ × min^−1^	24.2 ± 8.1	45	− 0.20	0.20
% predicted VO_2_ max	63.4 ± 16.0	45	− 0.08	0.61
Peak RER	1.2 ± 0.1	45	− 0.01	0.96
% predicted peak O_2_ pulse	77.2 ± 22.5	45	0.10	0.52
% predicted peak work rate	71.7 ± 20.5	44	− 0.17	0.28
Relative VO_2_ at VAT mL × kg^−1^ × min^−1^	14.5 ± 4.2	45	− 0.10	0.50
%VAT/Predicted peak VO_2_	38.4 ± 9.5	45	0.03	0.85
VE/VCO_2_ slope	34.4 ± 7.4	45	− 0.27	0.07
Laboratory				
Creatinine, mg/dL	0.7 ± 0.2	88	− 0.03	0.79
Albumin, mg/dL	4.4 ± 0.6	83	0.09	0.44
Absolute Lymphocyte Count, K cells/μL	1.2 ± 0.8	22	− 0.27	0.24

Partial correlation was performed to control for changes in maximum thoracic duct diameter with age. Bold indicates a p-value < 0.05. *EDV* end-diastolic volume, *BSA* body surface area, *ESV* end-systolic volume, *EF* ejection fraction, *APC* aortopulmonary collateral burden, *AVVR* atrioventricular valve regurgitation, *echo* echocardiography, *PVR* pulmonary vascular resistance, *EDP* end-diastolic pressure, *RER* respiratory exchange ratio, *VO*_*2*_ oxygen consumption, *VAT* ventilatory anaerobic threshold, *VE* ventilation

**Table 6:** Inter- and intraobserver agreement (n = 50)

	Interobserver		Intraobserver	
	Percent agreement	Kappa (95% CI)	Percent agreement	Kappa (95% CI)
Thoracic duct visualization	88%	0.76 (0.58, 0.94)	86%	0.72 (0.53, 0.91)
Thoracic duct tortuosity	76%	0.67 (0.51, 0.84)	76%	0.68 (0.50, 0.86)
	Mean difference ± SD	ICC (95% CI)	Mean difference ± SD	ICC (95% CI)
Thoracic duct diameter	0.37 ± 0.65	0.78 (0.74, 0.81)	0.41 ± 0.88	0.64 (0.57, 0.70)

*CI* confidence interval, *SD* standard deviation, *ICC* intraclass correlation coefficient

## Discussion

This study represents analysis of the thoracic duct on 3D bSSFP images from a large cohort of patients with a Fontan circulation. It is novel in exploring the clinical relevance of thoracic duct tortuosity in addition to its size. This study confirms that the thoracic duct is well visualized in patients with Fontan circulation on a 3D bSSFP sequence, with complete or nearly complete visualization in roughly two-thirds of cases. The thoracic duct was larger in patients with Fontan circulation relative to patients with rTOF. In patients with Fontan circulation, both larger thoracic duct maximum diameter and more tortuous thoracic ducts were associated with clinical outcomes. This study makes progress in increasing the utility of the 3D bSSFP sequence to evaluate the lymphatic system.

A lower incidence of thoracic duct visualization in patients with rTOF compared to patients with Fontan circulation may be due to the smaller maximum thoracic duct diameter in that population. In patients with Fontan circulation, larger maximum thoracic duct diameter was present in patients with PLE and correlated with a lower ejection fraction. PLE and plastic bronchitis are disorders related to lymphatic transit. As such, a larger thoracic duct size may be expected and this correlation with PLE is consistent with a prior study which found that thoracic duct diameter is larger in patients with Fontan circulation with concomitant PLE or plastic bronchitis [1]. The current study was likely underpowered to detect a relationship between thoracic duct features and plastic bronchitis (only two out of the 189 patients with Fontan circulation had plastic bronchitis). The correlation with lower ejection fraction was mild but may highlight an interaction between abnormal ventricular mechanics and disordered lymphatic production.

Although the thoracic duct has previously been demonstrated to be more tortuous in patients with Fontan circulation relative to healthy controls [11], this is the first study to be adequately powered to assess if tortuosity is associated with clinical outcomes. More tortuous thoracic ducts were present in patients with a higher absolute lymphocyte count. As chyle is rich in lymphocytes, a higher absolute lymphocyte count in patients with increased thoracic duct tortuosity may be related to increased lymphatic production. More tortuous thoracic ducts were also present in patients with NYHA class ≥ II. The correlation of increased tortuosity with NYHA class ≥ II is probably a result of exacerbation of disordered lymphatic drainage in the setting of Fontan circulation. One hypothesis is that disordered lymphatic drainage is due to increased central venous pressure. Although there was not a statistically significant association between thoracic duct tortuosity and Fontan pressure, our study may have been underpowered to detect this as only 45 patients had Fontan pressures available by catheterization. Finally, patients with more tortuous thoracic ducts had a higher indexed ventricular end-systolic volume. A higher end-systolic volume may indicate an interaction between abnormal ventricular mechanics and disordered lymphatic production.

### Limitations

There are a number of limitations to our study. A portion of the clinical events occurred prior to the CMR; hence, the thoracic duct variables studied should be viewed as correlations and not predictors of clinical outcomes. Similarly, hazard of composite outcome from the time of CMR could not be performed. The use of CMR to visualize the thoracic duct also biases the sample as patients with pacemakers and ICDs will be excluded, and sicker patients are more likely to be referred for CMR. Additionally, given the T2 prep pulse of 3D bSSFP sequences, the presence of pleural or pericardial effusions may make it harder to distinguish lymphatic channels. Also, in patients with venous decompressing collaterals it may be difficult to distinguish lymphatic channels from venous channels. Thoracic duct visualization and size is affected by Fontan hemodynamics on the day of the CMR examination and patient diet preceding the examination. As only moderate agreement on qualitative assessment of thoracic duct tortuosity was noted, quantitative assessments of thoracic duct tortuosity may be useful for future assessments [11].

## Conclusion

The thoracic duct can be well visualized in patients with Fontan circulation on a 3D bSSFP sequence. Larger thoracic duct diameter is associated with PLE and increased thoracic duct tortuosity is associated with an NYHA class ≥ II. Future studies can assess whether these thoracic duct characteristics affect clinical prognosis and whether management changes should be considered when abnormal thoracic duct characteristics are visualized.

## Supplementary Information

**Additional file 1. Table S1.** Characteristics of patients with Fontan circulation meeting inclusion criteria. **Table S2.** Demographics of patients with Fontan circulation by image visualization.

## Data Availability

The datasets used and/or analyzed during the current study are available from the corresponding author on reasonable request.

## Abbreviations

T2W: T2-weighted
3D-bSSFP: 3D balanced steady-state free precession
CMR: Cardiovascular magnetic resonance imaging
TD: Thoracic duct
rTOF: Repaired tetralogy of Fallot
NYHA: New York Heart Association
IQR: Interquartile range
BSA: Body surface area
PLE: Protein-losing enteropathy
SD: Standard deviation
SE: Standard error
