# Supplementary material for: Magnetic resonance three-dimensional steady-state free precession imaging of the thoracic duct in patients with Fontan circulation and its relationship to outcomes
Source: J Cardiovasc Magn Reson. 2023 Jun 12;25:28. doi: 10.1186/s12968-023-00937-w (PMC10258944; doi:10.1186/s12968-023-00937-w)
Supplement: Supplementary file 1 — Additional file 1. Table S1. Characteristics of patients with Fontan circulation meeting inclusion criteria. Table S2. Demographics of patients with Fontan circulation by image visualization. [file 12968_2023_937_MOESM1_ESM.docx]

**Table S1: Characteristics of patients with Fontan circulation meeting inclusion criteria (N=189)**

| Primary cardiac diagnosis |  |
| --- | --- |
| Tricuspid atresia | 34 (18%) |
| HLHS | 62 (33%) |
| DILV | 18 (10%) |
| DORV | 32 (17%) |
| Unbalanced CAVC | 13 (7%) |
| Small right heart | 10 (5%) |
| Other | 20 (11%) |
| Heterotaxy syndrome |  |
| Yes | 37 (20%) |
| No | 152 (80%) |
| Median age at Fontan, yrs. | 2.7 (2.2, 3.7) |
| Fontan type |  |
| Lateral tunnel | 125 (66%) |
| Extracardiac | 46 (24%) |
| RA-PA | 14 (7%) |
| Other | 4 (2%) |
| Fontan revision |  |
| Lateral tunnel | 3 (2%) |
| Extracardiac | 2 (4%) |
| RA-PA | 1 (7%) |
| Other | 2 (50%) |
| Ventricular morphology |  |
| Right | 89 (49%) |
| Left | 65 (36%) |
| Mixed/indeterminate | 27 (15%) |

**Table S1**: Values are count (%) unless otherwise specified. HLHS: hypoplastic left heart syndrome, DILV: double inlet left ventricle, DORV: double outlet right ventricle, CAVC: complete atrioventricular canal, RA: right atrium, PA: pulmonary artery.

**Table S2: Demographics of patients with Fontan circulation by image visualization (N=189)**

| Variable | Complete/Nearly complete  (n=122) | Incomplete/Not visualized  (n=67) | p-value |
| --- | --- | --- | --- |
| Male | 86 (71%) | 35 (52%) | **0.02** |
| Primary cardiac diagnosis |  |  | 0.21 |
| Tricuspid atresia | 19 (16%) | 15 (22%) |  |
| HLHS | 46 (38%) | 16 (24%) |  |
| DILV | 12 (10%) | 6 (9%) |  |
| DORV | 18 (15%) | 14 (21%) |  |
| Unbalanced CAVC | 8 (7%) | 5 (8%) |  |
| Small right heart | 4 (3%) | 6 (9%) |  |
| Other | 15 (12%) | 5 (8%) |  |
| Heterotaxy syndrome |  |  | 0.57 |
| Yes | 22 (18%) | 15 (22%) |  |
| No | 100 (82%) | 52 (78%) |  |
| Age at Fontan, yrs. |  |  |  |
| Median (IQR) | 2.7 (2.2, 3.5) | 2.9 (2.2, 4.1) | 0.60 |
| Fontan Type |  |  | 0.82 |
| Lateral tunnel | 82 (67%) | 43 (64%) |  |
| Extracardiac | 30 (25%) | 16 (24%) |  |
| RA-PA | 8 (7%) | 6 (9%) |  |
| Other | 2 (2%) | 2 (3%) |  |
| Fontan revision |  |  | 0.71 |
| Yes | 6 (5%) | 2 (3%) |  |
| No | 116 (95%) | 65 (97%) |  |
| Ventricular morphology |  |  | 0.27 |
| Right | 59 (50%) | 30 (48%) |  |
| Left | 39 (33%) | 26 (42%) |  |
| Both | 21 (18%) | 6 (10%) |  |
| Age at MRI, yrs. |  |  |  |
| Median (IQR) | 16.5 (10.9, 22.6) | 14.2 (11.1, 23.3) | 0.83 |
| BSA at MRI |  |  |  |
| Mean ± SD | 1.50 ± 0.44 | 1.49 ± 0.45 | 0.88 |

**Table S2:** Values are count (%), median (IQR), or mean ± standard deviation. HLHS: hypoplastic left heart syndrome, DILV: double inlet left ventricle, DORV: double outlet right ventricle, CAVC: complete atrioventricular canal, RA: right atrium, PA: pulmonary artery, BSA: body surface area.
